# Bis{*N*-benzyl-*N*-[2-(thio­phen-2-yl)eth­yl]di­thio­carbamato-κ^2^ *S*,*S*′}lead(II)

**DOI:** 10.1107/S1600536813019259

**Published:** 2013-07-17

**Authors:** E. Sathiyaraj, S. Thirumaran, B. Sridhar, S. Selvanayagam

**Affiliations:** a Department of Chemistry, Annamalai University, Annamalainagar 608 002, India; b Laboratory of X-ray Crystallography, Indian Institute of Chemical Technology, Hyderabad 500 007, India; c Department of Physics, Kalasalingam University, Krishnankoil 626 126, India

## Abstract

The mol­ecule of the title compound, [Pb(C_14_H_14_NS_3_)_2_], is located on a twofold rotation axis. The di­thio­carbamate anion *S*,*S*′-chelates to the Pb^II^ atom, which shows a Ψ-trigonal–bipyramidal coordination. The thio­phene ring is disordered over two positions, the major component having 71.3 (7)% occupancy. The mol­ecular conformation is stabilized by intra­molecular C—H⋯S inter­actions.

## Related literature

For a related structure, see: Sathiyaraj *et al.* (2012 ▶). For the superposition of structures, see: Gans & Shalloway (2001 ▶).
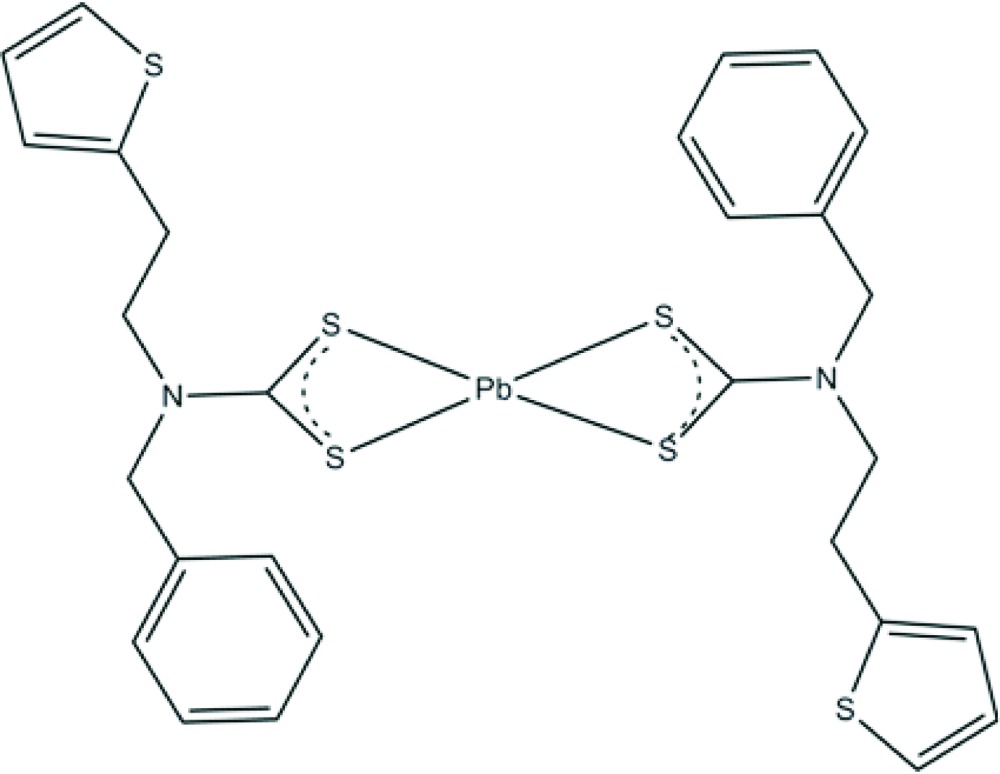


## Experimental

### 

#### Crystal data

[Pb(C_14_H_14_NS_3_)_2_]
*M* *_r_* = 792.07
Monoclinic, 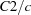

*a* = 27.459 (2) Å
*b* = 5.5580 (4) Å
*c* = 19.4670 (15) Å
β = 100.168 (2)°
*V* = 2924.3 (4) Å^3^
*Z* = 4
Mo *K*α radiation
μ = 6.22 mm^−1^
*T* = 292 K
0.20 × 0.08 × 0.06 mm

#### Data collection

Bruker SMART APEX CCD area-detector diffractometer
Absorption correction: multi-scan (*SADABS*; Bruker, 2001 ▶) *T*_min_ = 0.699, *T*_max_ = 0.707
16187 measured reflections
3487 independent reflections
3089 reflections with *I* > 2σ(*I*)
*R*_int_ = 0.023

#### Refinement

*R*[*F* ^2^ > 2σ(*F* ^2^)] = 0.026
*wR*(*F* ^2^) = 0.074
*S* = 1.01
3487 reflections
172 parameters
23 restraints
H-atom parameters constrained
Δρ_max_ = 0.91 e Å^−3^
Δρ_min_ = −0.84 e Å^−3^

### 

Data collection: *SMART* (Bruker, 2001 ▶); cell refinement: *SAINT* (Bruker, 2001 ▶); data reduction: *SAINT*; program(s) used to solve structure: *SIR92* (Altomare *et al.*, 1993 ▶); program(s) used to refine structure: *SHELXL2013* (Sheldrick, 2008 ▶); molecular graphics: *ORTEP-3 for Windows* (Farrugia, 2012 ▶) and *PLATON* (Spek, 2009 ▶); software used to prepare material for publication: *SHELXL2013* and *PLATON* (Spek, 2009) ▶.

## Supplementary Material

Crystal structure: contains datablock(s) I, global. DOI: 10.1107/S1600536813019259/ng5336sup1.cif

Structure factors: contains datablock(s) I. DOI: 10.1107/S1600536813019259/ng5336Isup2.hkl

Additional supplementary materials:  crystallographic information; 3D view; checkCIF report

## Figures and Tables

**Table 1:** Hydrogen-bond geometry (Å, °)

*D*—H⋯*A*	*D*—H	H⋯*A*	*D*⋯*A*	*D*—H⋯*A*
C2—H2*A*⋯S1	0.97	2.47	2.998 (4)	114
C8—H8*B*⋯S2	0.97	2.53	2.988 (4)	109

**Fig. 1.**
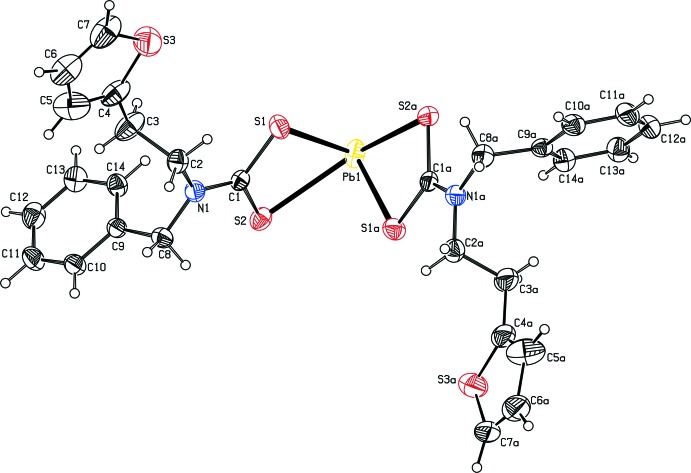
The molecular structure of the title compound, showing the atom-numbering scheme. Displacement ellipsoids are drawn at the 30% probability level. The minor occupied atoms of the disordered part have been omitted for clarity

## supplementary crystallographic information

### Comment

In continuation of our work on the crystal structure analysis of lead complexes, we have undertaken a single-crystal X-ray diffraction study for the title compound, and the results are presented here.

The X-ray study confirmed the molecular structure and atomic connectivity for (I), as illustrated in Fig. 1. The Pb atom is coordinated by four sulfur atom from two dithiocarbamate anions. The asymmetry in Pb-S bonds suggests that the lone pair on Pb(II) is stereochemically active. The geometry of this coordination PbS_4_ polyhedron is trigonal bipyramid. The geometry is similar to that reported for bis[N-benzyl-N-(2-phenylethyl)dithiocarbamato]lead(II) (Sathiyaraj *et al.*, 2012).The superposition of the coordination polyhedron (PbS_4_) of (I) with this related reported structure, using Qmol (Gans & Shalloway, 2001) shows the r.m.s. deviation is 0.005 Å.

The sum of the angles at N1 [359.7°] is in accordance with sp^2^ hybridization. The thiophene ring is disordered over two positions, with a major component being 71.3 (7)%. The dihedral angles between the phenyl and the major and minor components of the thiophene ring are 74.8 (1) and 74.7 (1)°, respectively.

In addition to the van der Waals interactions, the molecular structure is influenced only by intramolecular C—H···S hydrogen bonds.

### Experimental

Benzyl(2-(thiophene-2-yl)ethyl)amine (4 mmol) and carbon disulfide (4 mmol) were dissolved in ethanol (20ml) and stirred for 30minutes. To this solution, an aqueous solution (100 ml) of Pb(NO_3_)_2_ (2 mmol) was added with constant stirring. A pale yellow powder precipitated that was filtered and dried. Single crystals of (I) were obtained by slow evaporation of dichloromethane and acetone (1:1) solution of the title compound at room temperature.

### Refinement

H atoms were placed in idealized positions and allowed to ride on their parent atoms, with C—H distances of 0.93-0.97 Å, and Uiso(H) = 1.2U_eq_(C) for H atoms. The thiophene ring is disordered over two positions, with a major component being 71.3 (7)%. Pairs of C—S, C—C and C=C bond distances were restrained to 1.74 (1), 1.43 (1) and 1.37 (1) Å, respectively. The bond distances C3—C4 and C3—C4' were restrained to within 0.01 Å of each other. The temperature factors of C5' was set to those of S3 (as were these pairs: C4' to C4, S3' to C5, C6' to C6 and C7' to C7). The planarity of thiophene ring atoms were restrained to within 0.01 Å^3^ of each other. Pairs of C—S and C—C 1,3 bond distances were restrained to the values of 2.58 (1) and 2.33 (1) Å, respectively.

### Crystal data

**Table:**

[Pb(C_14_H_14_NS_3_)_2_]	*F*(000) = 1552
*M**_r_* = 792.07	*D*_x_ = 1.799 Mg m^−^^3^
Monoclinic, *C*2/*c*	Mo *K*α radiation, λ = 0.71073 Å
*a* = 27.459 (2) Å	Cell parameters from 8428 reflections
*b* = 5.5580 (4) Å	θ = 2.5–27.6°
*c* = 19.4670 (15) Å	µ = 6.22 mm^−^^1^
β = 100.168 (2)°	*T* = 292 K
*V* = 2924.3 (4) Å^3^	Needle, yellow
*Z* = 4	0.20 × 0.08 × 0.06 mm

### Data collection

**Table:**

Bruker SMART APEX CCD area-detector diffractometer	3089 reflections with *I* > 2σ(*I*)
Radiation source: fine-focus sealed tube	*R*_int_ = 0.023
ω scans	θ_max_ = 28.0°, θ_min_ = 2.1°
Absorption correction: multi-scan (*SADABS*; Bruker, 2001)	*h* = −35→35
*T*_min_ = 0.699, *T*_max_ = 0.707	*k* = −7→7
16187 measured reflections	*l* = −25→25
3487 independent reflections	

### Refinement

**Table:**

Refinement on *F*^2^	23 restraints
Least-squares matrix: full	Hydrogen site location: inferred from neighbouring sites
*R*[*F*^2^ > 2σ(*F*^2^)] = 0.026	H-atom parameters constrained
*wR*(*F*^2^) = 0.074	*w* = 1/[σ^2^(*F*_o_^2^) + (0.0483*P*)^2^ + 2.5197*P*] where *P* = (*F*_o_^2^ + 2*F*_c_^2^)/3
*S* = 1.01	(Δ/σ)_max_ = 0.001
3487 reflections	Δρ_max_ = 0.91 e Å^−^^3^
172 parameters	Δρ_min_ = −0.84 e Å^−^^3^

### Special details

**Table:**

Geometry. All esds (except the esd in the dihedral angle between two l.s. planes) are estimated using the full covariance matrix. The cell esds are taken into account individually in the estimation of esds in distances, angles and torsion angles; correlations between esds in cell parameters are only used when they are defined by crystal symmetry. An approximate (isotropic) treatment of cell esds is used for estimating esds involving l.s. planes.

### Fractional atomic coordinates and isotropic or equivalent isotropic displacement parameters (Å^2^)

**Table:**

	*x*	*y*	*z*	*U*_iso_*/*U*_eq_	Occ. (<1)
Pb1	0.5000	1.14399 (3)	0.7500	0.06000 (9)	
S1	0.42993 (3)	0.80812 (17)	0.71606 (4)	0.0567 (2)	
S2	0.49150 (3)	0.95155 (16)	0.61228 (4)	0.05250 (18)	
N1	0.42564 (10)	0.5970 (5)	0.59289 (13)	0.0471 (5)	
C1	0.44701 (10)	0.7691 (6)	0.63555 (14)	0.0422 (5)	
C2	0.38975 (13)	0.4246 (6)	0.61323 (18)	0.0540 (7)	
H2A	0.3901	0.4389	0.6630	0.065*	
H2B	0.4000	0.2623	0.6042	0.065*	
C3	0.33791 (14)	0.4638 (10)	0.5748 (3)	0.0812 (13)	
H3A	0.3287	0.6302	0.5805	0.097*	
H3B	0.3370	0.4356	0.5255	0.097*	
S3	0.28129 (10)	0.3223 (5)	0.67259 (11)	0.0953 (8)	0.713 (7)
C4	0.3015 (4)	0.3061 (14)	0.5993 (4)	0.0749 (18)	0.713 (7)
C5	0.2819 (4)	0.1084 (14)	0.5602 (4)	0.098 (2)	0.713 (7)
H5	0.2893	0.0692	0.5168	0.118*	0.713 (7)
C6	0.2487 (2)	−0.0298 (10)	0.5950 (4)	0.087 (2)	0.713 (7)
H6	0.2320	−0.1674	0.5766	0.105*	0.713 (7)
C7	0.2449 (3)	0.0673 (15)	0.6581 (4)	0.096 (3)	0.713 (7)
H7	0.2256	0.0057	0.6887	0.115*	0.713 (7)
S3'	0.2817 (3)	0.0438 (10)	0.5716 (4)	0.098 (2)	0.287 (7)
C4'	0.3056 (10)	0.273 (4)	0.5972 (14)	0.0749 (18)	0.287 (7)
C5'	0.2874 (9)	0.372 (3)	0.6629 (9)	0.0953 (8)	0.287 (7)
H5'	0.2978	0.5133	0.6868	0.114*	0.287 (7)
C6'	0.2511 (6)	0.204 (3)	0.6800 (6)	0.087 (2)	0.287 (7)
H6'	0.2344	0.2224	0.7174	0.105*	0.287 (7)
C7'	0.2443 (6)	0.015 (3)	0.6342 (9)	0.096 (3)	0.287 (7)
H7'	0.2226	−0.1114	0.6364	0.115*	0.287 (7)
C8	0.44094 (12)	0.5488 (6)	0.52616 (16)	0.0519 (7)	
H8A	0.4392	0.3766	0.5180	0.062*	
H8B	0.4753	0.5963	0.5298	0.062*	
C9	0.41122 (7)	0.6731 (3)	0.46313 (8)	0.0426 (6)	
C10	0.41287 (8)	0.5775 (3)	0.39761 (10)	0.0541 (7)	
H10	0.4307	0.4373	0.3936	0.065*	
C11	0.38794 (9)	0.6913 (4)	0.33807 (8)	0.0647 (9)	
H11	0.3890	0.6273	0.2942	0.078*	
C12	0.36135 (9)	0.9008 (4)	0.34405 (9)	0.0665 (9)	
H12	0.3447	0.9769	0.3042	0.080*	
C13	0.35970 (8)	0.9964 (3)	0.40957 (11)	0.0597 (8)	
H13	0.3419	1.1366	0.4136	0.072*	
C14	0.38463 (8)	0.8826 (3)	0.46911 (8)	0.0513 (7)	
H14	0.3835	0.9466	0.5129	0.062*	

### Atomic displacement parameters (Å^2^)

**Table:**

	*U*^11^	*U*^22^	*U*^33^	*U*^12^	*U*^13^	*U*^23^
Pb1	0.06332 (13)	0.04215 (11)	0.06445 (13)	0.000	−0.01642 (8)	0.000
S1	0.0622 (5)	0.0666 (5)	0.0418 (4)	−0.0148 (4)	0.0101 (3)	−0.0066 (3)
S2	0.0442 (4)	0.0575 (4)	0.0549 (4)	−0.0057 (3)	0.0064 (3)	0.0094 (3)
N1	0.0475 (13)	0.0506 (13)	0.0428 (12)	−0.0029 (10)	0.0064 (10)	−0.0014 (10)
C1	0.0369 (12)	0.0451 (14)	0.0425 (13)	0.0017 (11)	0.0009 (10)	0.0046 (12)
C2	0.0555 (17)	0.0498 (16)	0.0543 (17)	−0.0029 (14)	0.0028 (14)	0.0007 (14)
C3	0.0510 (19)	0.089 (3)	0.101 (3)	0.0012 (19)	0.0061 (19)	0.044 (3)
S3	0.0902 (14)	0.1277 (18)	0.0723 (11)	−0.0234 (11)	0.0257 (9)	0.0052 (10)
C4	0.046 (2)	0.079 (4)	0.098 (3)	0.007 (3)	0.009 (2)	0.036 (3)
C5	0.103 (2)	0.046 (3)	0.157 (4)	−0.014 (2)	0.054 (3)	−0.011 (3)
C6	0.070 (3)	0.071 (4)	0.119 (6)	−0.005 (3)	0.011 (4)	0.031 (4)
C7	0.062 (3)	0.120 (6)	0.103 (7)	−0.022 (3)	0.011 (4)	0.056 (6)
S3'	0.103 (2)	0.046 (3)	0.157 (4)	−0.014 (2)	0.054 (3)	−0.011 (3)
C4'	0.046 (2)	0.079 (4)	0.098 (3)	0.007 (3)	0.009 (2)	0.036 (3)
C5'	0.0902 (14)	0.1277 (18)	0.0723 (11)	−0.0234 (11)	0.0257 (9)	0.0052 (10)
C6'	0.070 (3)	0.071 (4)	0.119 (6)	−0.005 (3)	0.011 (4)	0.031 (4)
C7'	0.062 (3)	0.120 (6)	0.103 (7)	−0.022 (3)	0.011 (4)	0.056 (6)
C8	0.0540 (16)	0.0537 (17)	0.0485 (15)	0.0076 (14)	0.0106 (13)	−0.0070 (14)
C9	0.0415 (13)	0.0436 (14)	0.0439 (14)	−0.0071 (11)	0.0109 (11)	−0.0057 (11)
C10	0.0554 (17)	0.0604 (18)	0.0488 (16)	−0.0032 (14)	0.0157 (14)	−0.0080 (14)
C11	0.071 (2)	0.084 (3)	0.0416 (16)	−0.0063 (19)	0.0158 (16)	−0.0065 (16)
C12	0.066 (2)	0.081 (2)	0.0514 (18)	−0.0083 (18)	0.0062 (16)	0.0150 (17)
C13	0.0651 (19)	0.0493 (17)	0.0624 (19)	−0.0014 (15)	0.0049 (16)	0.0041 (15)
C14	0.0577 (18)	0.0453 (16)	0.0498 (16)	−0.0026 (13)	0.0062 (14)	−0.0076 (12)

### Geometric parameters (Å, º)

**Table:**

Pb1—S1^i^	2.6785 (9)	C7—H7	0.9300
Pb1—S1	2.6785 (9)	S3'—C4'	1.48 (2)
Pb1—S2	2.8575 (9)	S3'—C7'	1.733 (9)
Pb1—S2^i^	2.8576 (9)	C4'—C5'	1.56 (3)
S1—C1	1.727 (3)	C5'—C6'	1.446 (9)
S2—C1	1.709 (3)	C5'—H5'	0.9300
N1—C1	1.333 (4)	C6'—C7'	1.369 (9)
N1—C8	1.459 (4)	C6'—H6'	0.9300
N1—C2	1.478 (4)	C7'—H7'	0.9300
C2—C3	1.502 (5)	C8—C9	1.515 (4)
C2—H2A	0.9700	C8—H8A	0.9700
C2—H2B	0.9700	C8—H8B	0.9700
C3—C4	1.471 (6)	C9—C10	1.3900
C3—C4'	1.497 (10)	C9—C14	1.3900
C3—H3A	0.9700	C10—C11	1.3900
C3—H3B	0.9700	C10—H10	0.9300
S3—C4	1.622 (7)	C11—C12	1.3900
S3—C7	1.728 (7)	C11—H11	0.9300
C4—C5	1.389 (8)	C12—C13	1.3900
C5—C6	1.449 (7)	C12—H12	0.9300
C5—H5	0.9300	C13—C14	1.3900
C6—C7	1.361 (7)	C13—H13	0.9300
C6—H6	0.9300	C14—H14	0.9300
			
S1^i^—Pb1—S1	91.64 (5)	S3—C7—H7	125.1
S1^i^—Pb1—S2	84.65 (2)	C4'—S3'—C7'	97.1 (11)
S1—Pb1—S2	64.63 (2)	S3'—C4'—C3	140 (2)
S1^i^—Pb1—S2^i^	64.63 (2)	S3'—C4'—C5'	113.3 (10)
S1—Pb1—S2^i^	84.65 (2)	C3—C4'—C5'	106.3 (15)
S2—Pb1—S2^i^	136.04 (3)	C6'—C5'—C4'	107.1 (7)
C1—S1—Pb1	90.74 (10)	C6'—C5'—H5'	126.4
C1—S2—Pb1	85.25 (10)	C4'—C5'—H5'	126.4
C1—N1—C8	121.5 (3)	C7'—C6'—C5'	111.3 (6)
C1—N1—C2	122.7 (3)	C7'—C6'—H6'	124.3
C8—N1—C2	115.5 (3)	C5'—C6'—H6'	124.3
N1—C1—S2	121.2 (2)	C6'—C7'—S3'	111.1 (7)
N1—C1—S1	119.7 (2)	C6'—C7'—H7'	124.4
S2—C1—S1	119.17 (18)	S3'—C7'—H7'	124.4
N1—C2—C3	113.0 (3)	N1—C8—C9	116.0 (2)
N1—C2—H2A	109.0	N1—C8—H8A	108.3
C3—C2—H2A	109.0	C9—C8—H8A	108.3
N1—C2—H2B	109.0	N1—C8—H8B	108.3
C3—C2—H2B	109.0	C9—C8—H8B	108.3
H2A—C2—H2B	107.8	H8A—C8—H8B	107.4
C4—C3—C2	113.0 (5)	C10—C9—C14	120.0
C4'—C3—C2	108.1 (12)	C10—C9—C8	117.94 (16)
C4—C3—H3A	109.0	C14—C9—C8	122.00 (16)
C2—C3—H3A	109.0	C11—C10—C9	120.0
C4—C3—H3B	109.0	C11—C10—H10	120.0
C2—C3—H3B	109.0	C9—C10—H10	120.0
H3A—C3—H3B	107.8	C12—C11—C10	120.0
C4—S3—C7	95.1 (3)	C12—C11—H11	120.0
C5—C4—C3	120.9 (7)	C10—C11—H11	120.0
C5—C4—S3	111.5 (4)	C11—C12—C13	120.0
C3—C4—S3	127.6 (5)	C11—C12—H12	120.0
C4—C5—C6	112.2 (5)	C13—C12—H12	120.0
C4—C5—H5	123.9	C14—C13—C12	120.0
C6—C5—H5	123.9	C14—C13—H13	120.0
C7—C6—C5	111.4 (5)	C12—C13—H13	120.0
C7—C6—H6	124.3	C13—C14—C9	120.0
C5—C6—H6	124.3	C13—C14—H14	120.0
C6—C7—S3	109.8 (4)	C9—C14—H14	120.0
C6—C7—H7	125.1		
			
C8—N1—C1—S2	3.1 (4)	C7'—S3'—C4'—C3	−169 (4)
C2—N1—C1—S2	175.4 (2)	C7'—S3'—C4'—C5'	0.01 (16)
C8—N1—C1—S1	−176.5 (2)	C4—C3—C4'—S3'	132 (12)
C2—N1—C1—S1	−4.1 (4)	C2—C3—C4'—S3'	−102 (3)
Pb1—S2—C1—N1	−175.3 (2)	C4—C3—C4'—C5'	−38 (9)
Pb1—S2—C1—S1	4.30 (16)	C2—C3—C4'—C5'	88.1 (9)
Pb1—S1—C1—N1	175.0 (2)	S3'—C4'—C5'—C6'	0.0 (2)
Pb1—S1—C1—S2	−4.57 (17)	C3—C4'—C5'—C6'	173 (2)
C1—N1—C2—C3	110.3 (4)	C4'—C5'—C6'—C7'	0.0 (4)
C8—N1—C2—C3	−76.9 (4)	C5'—C6'—C7'—S3'	0.0 (5)
N1—C2—C3—C4	−175.1 (5)	C4'—S3'—C7'—C6'	0.0 (4)
N1—C2—C3—C4'	177.4 (13)	C1—N1—C8—C9	−94.7 (3)
C4'—C3—C4—C5	−48 (9)	C2—N1—C8—C9	92.4 (3)
C2—C3—C4—C5	−104.5 (6)	N1—C8—C9—C10	−159.4 (2)
C4'—C3—C4—S3	129 (10)	N1—C8—C9—C14	23.5 (4)
C2—C3—C4—S3	71.9 (10)	C14—C9—C10—C11	0.0
C7—S3—C4—C5	0.00 (15)	C8—C9—C10—C11	−177.2 (2)
C7—S3—C4—C3	−176.7 (11)	C9—C10—C11—C12	0.0
C3—C4—C5—C6	177.2 (10)	C10—C11—C12—C13	0.0
S3—C4—C5—C6	0.2 (2)	C11—C12—C13—C14	0.0
C4—C5—C6—C7	−0.4 (4)	C12—C13—C14—C9	0.0
C5—C6—C7—S3	0.4 (4)	C10—C9—C14—C13	0.0
C4—S3—C7—C6	−0.2 (3)	C8—C9—C14—C13	177.1 (2)

Symmetry code: (i) −*x*+1, *y*, −*z*+3/2.

### Hydrogen-bond geometry (Å, º)

**Table:**

*D*—H···*A*	*D*—H	H···*A*	*D*···*A*	*D*—H···*A*
C2—H2*A*···S1	0.97	2.47	2.998 (4)	114
C8—H8*B*···S2	0.97	2.53	2.988 (4)	109
